# Editorial: Impact of overall health on oral health of children and adolescents

**DOI:** 10.3389/fdmed.2025.1767198

**Published:** 2026-01-06

**Authors:** Alisa Lee, Sreekanth Kumar Mallineni, Rosalyn Sulyanto

**Affiliations:** 1 Department of Dentistry, Boston Children's Hospital, Boston, MA, United States; 2 Dr Sulaiman Al-Habib Medical Group, Riyadh, Saudi Arabia; 3 Department of Developmental Biology, School of Dental Medicine, Harvard University, Boston, MA, United States

**Keywords:** early childhood caries (ECC), health disparities, interdisciplinary care, oral-systemic health, pediatric dentistry, pediatric oral health, preventive dentistry, special health care needs children

Oral health plays a foundational role in children's overall health and development. The relationship among oral disease, nutrition, behavioral health, systemic conditions, and social determinants is multifaceted and interconnected (1). As oral diseases continue to affect billions worldwide, global health research demonstrates that the burden of oral disease is both widespread and deeply entrenched, particularly in vulnerable populations (1, 2). Early childhood caries (ECC), one of the most prevalent chronic diseases of childhood, remains strongly linked to growth, diet, and socioeconomic context (2, 3). Systemic infections and congenital conditions also frequently present with early oral manifestations, underscoring the central role of dental providers in early recognition, risk assessment, and interdisciplinary care (4, 5). Integrating dental and medical research is essential to understand oral-systemic interactions and improve child health outcomes.

The present research topic comprises eight articles, including case reports, systematic and narrative reviews, qualitative analyses, and interventional and utilization studies, that examine biological, nutritional, behavioral, and developmental determinants of pediatric oral health. Across varied settings, methodologies, and clinical populations, these studies have a common theme: oral health cannot be separated from systemic health, developmental outcomes, behavioral patterns, or health equity.

Articles in this collection highlight the importance of oral health clinicians in identifying early signs of systemic disease and collaborating with medical colleagues. Danesh et al. reported an adolescent with painful oral ulcerations during acute COVID-19 infection, diagnosed as reactive infectious mucocutaneous eruption limited to the oral mucosa. The case reinforces emerging evidence that SARS-CoV-2 can induce mucocutaneous changes (4) and underscores the importance of interdisciplinary management.

Articles in this collection have demonstrated the cyclical interaction between nutritional status and dental disease and the need for integrated public health strategies. In their rapid review, Aulia et al. synthesized the global evidence demonstrating a bidirectional relationship between growth stunting and ECC, with proposed mechanisms including malnutrition, chronic inflammation, feeding difficulty, and oral pain (6). Their findings underscore ECC as both a contributor to and a consequence of impaired childhood growth. Complementing these findings, Putri et al. reported that children with growth stunting demonstrated poorer oral hygiene practices and higher caries progression, reinforcing the need for early preventive oral health interventions within nutrition and growth-monitoring programs.

Craniofacial differences and congenital anomalies further illustrate oral-systemic connections. Lai et al. introduced a parent-implemented early intervention model for Mandarin-speaking infants and toddlers with cleft lip and/or palate, demonstrating improved early speech and language outcomes. The study illustrated how feeding, oral structure, and early communication skills intersect with caregiver involvement, emphasizing the role of family-centered care in optimizing language development.

Behavioral and lifestyle factors also play a critical role in oral disease risk. Excessive consumption of sugar-sweetened beverages (SSBs) remains a significant contributor to caries, obesity, and metabolic dysregulation (7). Yusuf and Valenzuela evaluated a school-based group motivational interviewing intervention designed to reduce SSB intake among adolescents. Consistent with existing behavioral science literature, their findings supported motivational interviewing as an effective behavioral strategy for promoting healthier behaviors in youth (8). The study highlighted the potential of school-based preventive strategies to address modifiable nutritional risks.

Financial and non-financial barriers also shape adolescents’ ability to access oral healthcare. In their qualitative investigation, Alsharif and Kassim explored how barriers such as payment methods and fear contribute to dental care non-attendance among adolescents. Their findings revealed that barriers were not solely financial but were also tied to patient fear and anxiety. This study highlighted the need for both cost-effective strategies and psychoeducational approaches to improve dental attendance.

Expanding on inequities among vulnerable populations, Tran et al. examined preventive dental service utilization among adolescents with developmental disabilities and obesity, finding that despite comparable preventive visit rates to their peers, these adolescents remain at disproportionately high risk for oral disease. This discrepancy suggests that standard preventive care may be insufficient in this population and reinforces longstanding evidence that children with special healthcare needs require tailored, coordinated, and interdisciplinary approaches to achieve optimal oral health (9).

Genetic and congenital conditions also shape oral health trajectories from early childhood. Morandini et al. provided a narrative review of ectodermal dysplasia (ED), detailing its clinical presentation, underlying genetic pathways, and implications for long-term medical and dental management. ED is a genetically and clinically complex condition in which oral findings are central. Understanding characteristic dental patterns and providing early, coordinated dental care are critical to improving both medical outcomes and quality of life for patients with ED. Their work illustrated how early diagnosis, multidisciplinary treatment planning, and psychosocial support can improve care and highlighted the need for integrated medical-dental models for complex genetic disorders (10).

Taken together, the eight articles in this research topic convey a clear message: pediatric oral health is fundamentally intertwined with systemic health, nutrition, development, behavior, and social context. The studies collectively position oral health as both an early indicator of broader health challenges and a critical point for intervention. By highlighting oral manifestations of infectious disease, the bidirectional relationship between nutrition and caries, the unmet needs of children with developmental disabilities, and the lifelong implications of genetic conditions, this collection underscores the importance of integrating dental, medical, and public health research to address the multifactorial determinants of oral health and reduce disparities across diverse pediatric populations. Overall, the research presented in this topic advances our understanding of oral-systemic health connections and identifies actionable pathways to improving child health globally.
